# Adalimumab Accounts for Long-Term Control of Noninfectious Uveitis Also in the Absence of Concomitant DMARD Treatment: A Multicenter Retrospective Study

**DOI:** 10.1155/2019/1623847

**Published:** 2019-02-10

**Authors:** Alice Bitossi, Alessandra Bettiol, Elena Silvestri, Gerardo Di Scala, Daniela Bacherini, Giuseppe Lopalco, Vincenzo Venerito, Florenzo Iannone, Antonio Vitale, Gian Marco Tosi, Domenico Prisco, Stanislao Rizzo, Claudia Fabiani, Luca Cantarini, Gianni Virgili, Lorenzo Vannozzi, Giacomo Emmi

**Affiliations:** ^1^Department of Surgery and Translational Medicine, Eye Clinic, University of Firenze, 50134, Italy; ^2^Department of Neurosciences, Psychology, Drug Research and Child Health (NEUROFARBA), University of Firenze, 50139, Italy; ^3^Department of Experimental and Clinical Medicine, University of Firenze, 50134, Italy; ^4^Rheumatology Unit, Department of Emergency and Organ Transplantation (DETO), University of Bari, 70121, Italy; ^5^Research Center of Systemic Autoinflammatory Diseases and Behçet's Disease and Rheumatology-Ophthalmology Collaborative Uveitis Center, Department of Medical Sciences, Surgery and Neurosciences, University of Siena, 53100, Italy; ^6^Ophthalmology Unit of the Department of Medicine, Surgery and Neuroscience, University of Siena, Siena 53100, Italy

## Abstract

**Objective:**

This study was aimed at assessing the long-term ocular control of adalimumab (ADA) in a large real-world population with noninfectious primary or secondary uveitis, focusing on the steroid-sparing effect and on disease-modifying antirheumatic drug (DMARD) cotreatment.

**Methods:**

In this retrospective, multicenter study, the efficacy of ADA was evaluated in terms of ocular control, changes in best-corrected visual acuity (BCVA), corticosteroid-sparing effect, and drug retention rate, overall and stratified according to DMARD cotreatment.

**Results:**

106 patients were included. 88.7% had an associated systemic disease. After 6 and 12 months, proportions of patients with effective ocular control were 83.7% and 83.3%, respectively. At last the follow-up, 94.6% of patients had satisfactory ocular control. No difference in terms of ocular control at all time points emerged among patients starting ADA for ocular *vs*. systemic involvements. Patients with poor baseline BCVA remained stable or improved, while those with good BCVA hardly worsened. At 6 and 12 months, the median dose of prednisone significantly reduced to 5 mg/day (0-5) and 2.5 mg/day (0-5) (*p* < 0.001). Over a median follow-up of 36 months, 38 subjects discontinued ADA treatment. Mild to moderate side effects were reported in 7 patients (6.6%). ADA ocular control, corticosteroid-sparing effect, and drug retention rate were not influenced by the concomitant use of DMARDs.

**Conclusion:**

The long-term ocular control of ADA in noninfectious primary or secondary uveitis is confirmed, also for BCVA preservation. Concomitant use of DMARDs does not provide additional benefits to ADA alone in terms of ocular control, steroid spare, and drug retention rate.

## 1. Introduction

Noninfectious primary or secondary uveitis is a group of vision-threatening diseases characterized by intraocular inflammation. It can occur as an isolated involvement of the eyes or associated with a systemic condition, including Behçet's syndrome (BS), juvenile idiopathic arthritis (JIA), rheumatoid arthritis (RA), Vogt-Koyanagi-Harada (VKH), sarcoidosis (SAR), ankylosing spondylitis (AS), psoriatic arthritis (PsA), inflammatory bowel disease (IBD), and multiple sclerosis [1, 2].

In the developed world, uveitis accounts for an estimated 10 to 15% of the cases of total blindness and up to 20% of legal blindness [1–3]. Uveitis can affect people of any age, but it most commonly develops in people between the ages of 20 and 59 years and is a major cause of visual morbidity in the working age group [2].

Corticosteroids are still the mainstay of treatment [1]. However, long-term use of moderate to high doses of corticosteroids can result in serious adverse events, including both ocular morbidity, such as glaucoma and cataract, and systemic adverse events, including impaired glucose tolerance, hypertension, osteoporosis, and infection susceptibility [2].

Other therapeutic options for noninfectious primary or secondary uveitis comprised traditional immunosuppressants (disease-modifying antirheumatic drugs (DMARDs)), such as cyclosporine (CsA), methotrexate (MTX), azathioprine (AZA), sulfasalazine (SSZ), and mycophenolate mofetil (MMF). However, a significant proportion of cases of uveitis cannot be controlled [4].

Thus, in recent years, there has been a great interest in identifying more effective, corticosteroid-sparing therapies, ideally targeting specific mediators of the immune response [5]. The proinflammatory cytokine tumor necrosis factor *α* (TNF-*α*) is thought to play a key role in uveitis inflammation, and aqueous humor and serum levels of TNF-*α* are upregulated in patients with uveitis [1–4, 6].

Adalimumab (ADA), a recombinant human immunoglobulin (IgG1) monoclonal antibody that specifically binds to TNF-*α* [2, 7, 8], is the only systemic noncorticosteroid agent currently approved for the treatment of noninfectious primary or secondary uveitis [9].

Indeed, two phase 3 clinical trials, VISUAL-1 and VISUAL-2, have been conducted among patients with active and inactive uveitis, respectively. In both trials, ADA led to a significant and clinical improvement in visual functioning [1, 8]. Furthermore, in the phase 3, open-label, extension trial VISUAL-III, ADA proved effective in inducing quiescence, improving best-corrected visual acuity (BCVA), and reducing the daily uveitis-related systemic steroid use, with poor safety concerns [10].

Nevertheless, a large proportion of subjects included in these trials had idiopathic uveitis, in the absence of systemic inflammatory disorders. Thus, the replicability of these results, and in particular of the steroid-sparing potential of ADA, in patients with uveitis secondary to a systemic disease, is still a matter of debate. What is more, the real contribution of DMARDs in the response to and drug retention rate on ADA treatment, particularly in secondary uveitis, is still unclear. Furthermore, only a small number of studies have evaluated ADA efficacy for the treatment of noninfectious primary or secondary uveitis in a real-world setting [11–13].

In light of these considerations, our primary objective was to assess the long-term ocular control of ADA in a large and heterogeneous real-world population with noninfectious primary or secondary uveitis. The secondary objectives of the study were the evaluation of ADA effectiveness in inducing changes in the BCVA and variations in corticosteroid use, the ADA retention rate and its safety profile, and the assessment of the impact of concomitant DMARD treatment on ocular response, steroid variations, and drug retention rate.

## 2. Materials and Methods

### 2.1. Study Setting and Population

A retrospective, observational, multicenter study was conducted based on medical chart review of 3 Italian tertiary referral centers (Firenze, Siena, and Bari). Ocular data recorded in the medical chart derived from either ambulatory ocular visits alone or association to a concomitant angiography, autofluorescence, or optic coherence tomography (OCT) examination.

All adult patients with noninfectious primary or secondary uveitis (anterior, intermediate, posterior uveitis, panuveitis or scleritis, idiopathic or secondary to a systemic disease, treated with ADA standard dose (40 mg/every 2 weeks subcutaneously)) were included.

We collected data on demographic information, systemic diagnosis (BS, JIA, PsA, AS, and IBD), type of ocular involvement, and previous or ongoing immune-modulating treatment.

We defined ocular flare as the presence of anterior chamber cells of 1+ (or higher) or vitreous haze of 1+ (or more) or active chorioretinal lesions, inflammatory retinal vascular lesions, or optic nerve inflammation, or by medical chart reviews, as previously described [12]. For patients with scleritis, ocular flare was defined as the presence of scleral inflammation of 1+ (or higher) or clinical and ultrasonographic findings as previously described [14, 15].

Evaluation was performed per patient and not per eye. When both eyes were affected, the worse eye was evaluated.

For each patient, clinical data were collected at the time of the first ADA administration, at 6 and 12 months following ADA beginning and at time of the last follow-up visit. No exclusion criterion was applied to follow-up, i.e., patients not reaching the 6 months of follow-up were not excluded from the study, thus including in the study the whole cohort of patients treated with adalimumab in our real clinical practice.

### 2.2. Outcomes

The primary endpoint was ADA ocular control at 6 and 12 months and at last available follow-up. Ocular control was defined as follows: absence of ocular flare in both eyes and reduction of the daily prednisone (or prednisone-equivalent) dose to ≤10 mg/day or halving of the initial steroid dose.

Secondary outcomes included (i) changes in BCVA, (ii) variations in corticosteroids' daily dose, (iii) drug retention rate, (iv) occurrence of any adverse event, and (v) impact of concomitant DMARD treatment on ocular response, steroid variations, and drug retention rate.

As for BCVA, subjects were categorized into three groups, based on the BCVA of the worst eye: VA ≤20/200, >20/200-≤20/40, and >20/40 (Snellen chart). Variations in VA were defined as the proportion of subjects that changed VA group from baseline to final evaluation.

As for DMARD cotreatment, subjects were categorized in the “ADA+DMARD” group, if both ADA and DMARDs were administered during the entire first year of available follow-up, and the “only ADA” group, if DMARDs were never administered during the entire first year of available follow-up. Subjects cotreated with DMARDs only for a certain period during follow-up were excluded from this analysis.

### 2.3. Statistical Analysis

Descriptive statistics was used to describe the demographic and clinical characteristics of the included subjects. Continuous variables were reported as median values and relative interquartile ranges (IQR) and were compared using the Kruskal-Wallis test. Categorical variables were reported as absolute frequencies and percentages and were compared using the Fisher exact test. Univariate Cox regression model was used to derive Kaplan-Meier curves for drug retention rate on treatment. Statistical analysis was performed using the Stata software version 14. Statistical significance was considered for *p* values < 0.05.

## 3. Results

### 3.1. Characteristics of Patients and Treatments

One hundred and six patients treated with ADA for noninfectious primary or secondary uveitis were included. Median age (IQR) at onset of uveitis was 29 years (20.5-40); fifty-nine patients (55.66%) were female. Median follow-up was 36.02 months (16.27-59.17). Clinical characteristics of the cohort are summarized in Table 1.

Fifty-one patients (48.11%) had intermediate uveitis associated with either posterior or panuveitis, 45 (42.45%) had anterior uveitis, and 10 (9.43%) had scleritis (alone or with uveitis); 64 patients (60.38%) had bilateral uveitis.

As for systemic involvement, the great majority of patients (94; 88.68%) had an associated systemic disease, whereas twelve patients had idiopathic uveitis (11.32%). The most common associated systemic conditions were BS (66, 62.26%), followed by JIA, PsA, AS, and IBD, which have been included in a single group named “other” (28, 26.42%: 11 JIA, 5 PsA, 11 AS, and 1 IBD).

76 patients began ADA for the treatment of the ocular involvement (associated with systemic manifestations in 49 of them), whereas 30 patients started ADA for the systemic manifestations, although all of them had history of ocular involvement. Specifically, 12 patients had ocular manifestations in the year before ADA beginning (of them, 3 patients had more than one ocular flare in the same year); the remaining 18 patients had history of ocular involvements but had no ocular flare in the year before ADA beginning_._

Specifically, out of the 49 subjects with both ocular and systemic involvements at the time of ADA beginning, 30 had BS, 8 had JIA, 8 had AS, and 3 had PsA. As for the 30 patients that started ADA for systemic manifestations, 21 had BS, 4 had JIA, 2 had PsA, 2 had AS, and 1 had IBD.

Before ADA beginning, 59 patients (55.66%) were treated with synthetic DMARDs, 7 (6.60%) with other biologics, 23 (21.70%) with both synthetic and biologic DMARDs, and 17 (16.04%) did not receive any systemic immune-modulating treatment. As for biologics, 17 patients had been treated with infliximab, 4 with etanercept, 2 with anakinra, 1 with rituximab, 1 with certolizumab, and 5 with more than one drug. As for DMARDs, 21 patients had been treated with CsA, 21 with MTX, 16 with AZA, 2 with SSZ, and 24 with more than one drug.

Otherwise, at the beginning of ADA treatment, 53 patients (50.00%) were treated with synthetic DMARDs, 7 (6.60%) with biologics, and 4 (3.77%) with both, whereas 31 patients (29.25%) were off-treatment.

As for biologics, 8 patients were treated with infliximab, 1 with etanercept, 1 with anakinra, and 1 with rituximab. As for DMARDs, 15 were treated with CsA, 23 with MTX, 17 with AZA, 1 with SSZ, and 1 with MMF.

### 3.2. Primary Endpoint: Ocular Control

Table 2 shows the ocular outcomes of ADA therapy over time, in terms of overall ocular control. Due to the variability in treatment length and to losses at follow-up, the number of observed patients decreased over time from 106 at baseline to 92 at 6 months and to 78 at 12 months.

Within 6 months of ADA treatment, ocular control, defined as reported in Materials and Methods, was achieved in 77 out of 92 patients (83.7%). Specifically, proportions of ocular control at 6 months were 83.1% among patients starting ADA for the ocular involvement (alone or with concomitant systemic manifestations; *n* = 54 out of 65) and 85.2% among patients starting ADA for the systemic involvement (*n* = 23 out of 27) (*p* = 1.000).

The proportion of subjects achieving ocular control was maintained at 12 months of treatment (65 out of 78 subjects; 83.3%). Specifically, proportions of ocular control at 12 months were 80.4% among patients starting ADA for the ocular involvement (*n* = 45 out of 56) and 90.9% among patients starting ADA for the systemic involvement (*n* = 23 out of 27) (*p* = 0.330).

Among the 55 patients with an available follow-up longer than 12 months (median follow-up was 36.02 months), this percentage further increased to 94.5%. Of note, proportions of ocular control among patients with an available follow-up longer than 12 months were 91.7% in patients with ocular involvement and 100.0% among patients with only systemic involvement at the time of ADA beginning.

Notably, 18 patients had macular edema (ME) at baseline (16.98%). Among them, ME resolved in 14 (77.78%) patients. Of note, in one patient diagnosed with scleritis, ME developed during ADA treatment.

During ADA treatment, eight patients required periocular steroid injections.

### 3.3. Secondary Endpoints

#### 3.3.1. Changes in Visual Acuity

**Figure 1:**
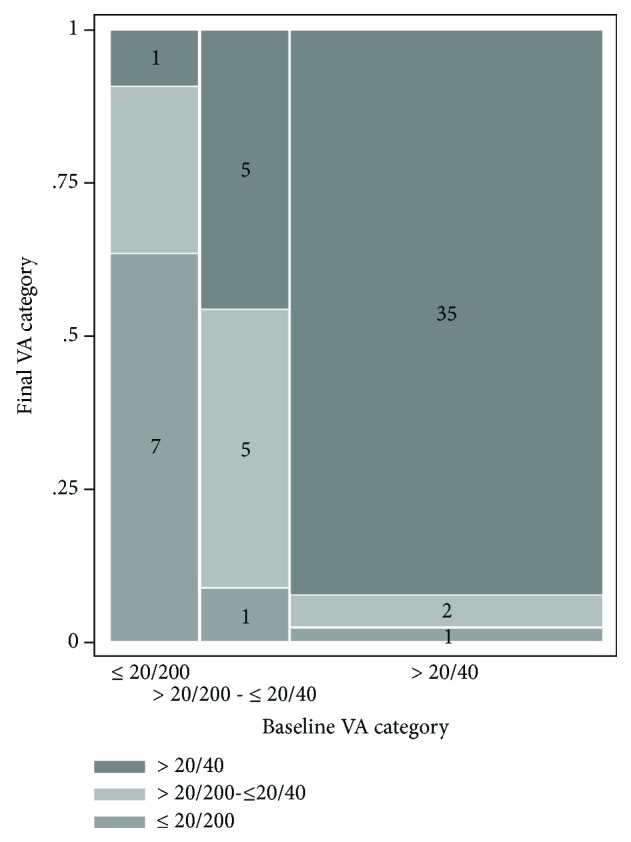
Changes in visual acuity. VA: visual acuity.

Data on VA were available for 60 subjects.  Figure 1 shows changes in VA, from baseline to final evaluation. At baseline, 11 subjects (18.33%) were included in the lowest VA category (≤20/200), 11 (18.33%) in the intermediate category (>20/200-≤20/40), and 38 (63.33%) in the highest category (>20/40).

Among subjects in the lowest category, 7 remained stable whereas 4 improved (with one patient improving to >20/40). Among subjects in the intermediate category, 5 preserved their VA and improvement to >20/40 occurred in another 5 cases; only 1 subject worsened to ≤20/200, due to the onset of cataract. Among the 38 subjects with the highest VA, 35 remained stable, whereas 3 subjects worsened and one of them decreased VA to ≤20/200. In two of these patients, worsening was related to the onset of cataract; the patient that worsened to VA ≤20/200 had scleritis and developed ME during ADA treatment.

#### 3.3.2. Corticosteroid-Sparing Effect

**Figure 2:**
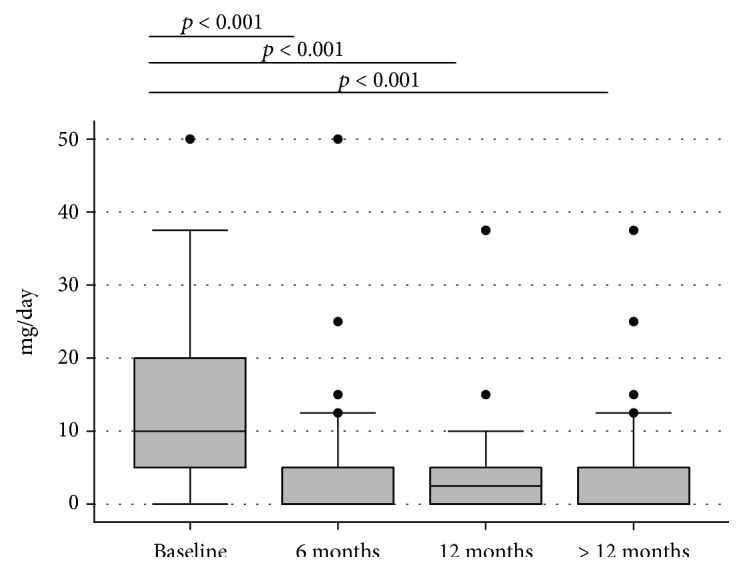
Variations of corticosteroid dosage over time.

The variation of corticosteroid dosage during ADA treatment is reported in Figure 2. Median prednisone dose at the time of ADA beginning was 10 (7.5-20) mg/day. At 6 months, median dose of prednisone was significantly reduced to 5 (0-5) mg/day (*p* < 0.001). At 12 months, the dosage further decreased to 2.5 (0-5) mg/day (*p* < 0.001), and for patients with a follow-up longer than 12 months, the median prednisone dose at last follow-up was 0 (0-5).

#### 3.3.3. Drug Retention Rate on Adalimumab Treatment

**Figure 3:**
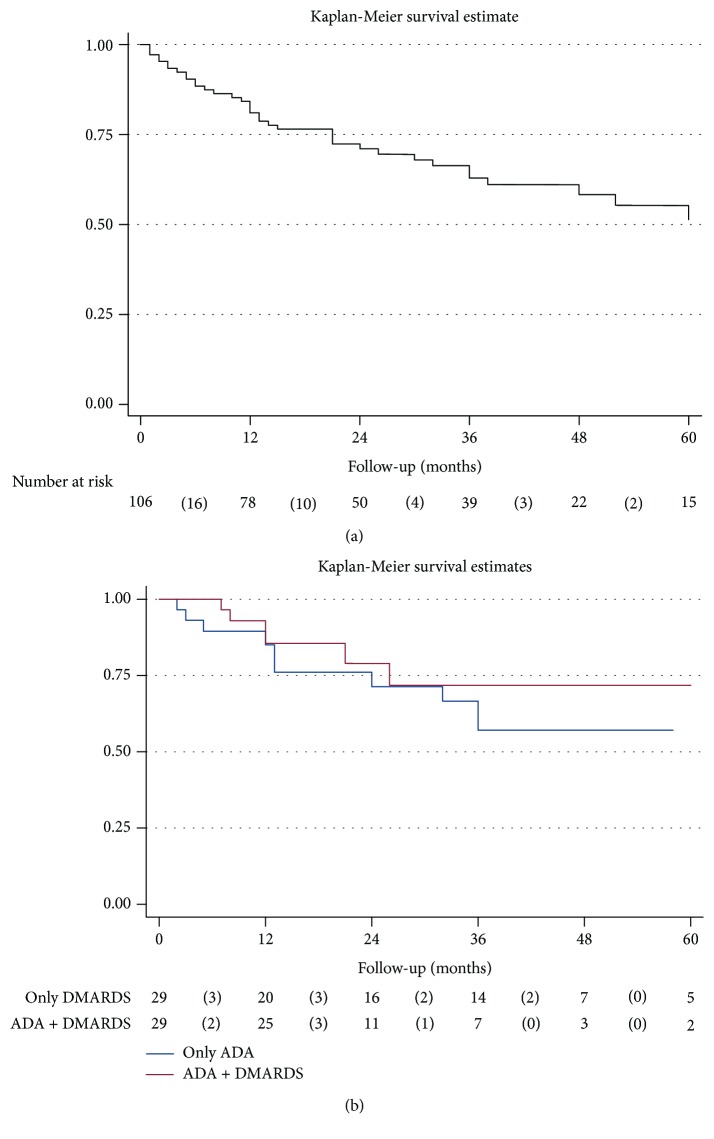
Drug retention rate on adalimumab treatment, overall (upper box) and stratified according to concomitant use of DMARDs vs. adalimumab in monotherapy (lower box). ADA: adalimumab; DMARDs: disease-modifying antirheumatic drugs.

Figure 3 shows the drug retention rate on ADA treatment. Over the total available follow-up, 38 subjects discontinued ADA. Rate of discontinuation was 0.15 per person-year (0.11–0.21). Most frequent reasons for discontinuation were inefficacy (*n* = 12), followed by loss of efficacy (*n* = 10), side effects (*n* = 7), ocular remission (*n* = 4), and pregnancy (*n* = 1).

#### 3.3.4. Safety

Seven patients had mild to moderate side effects. In detail, one case reported recurrent infections, one back eczema, one vertigo, and one pneumonia, whereas in the remaining 3 cases, the exact adverse effect was not specified. All seven side effects required treatment discontinuation.

#### 3.3.5. Impact of DMARD Cotreatment on Ocular Response, Corticosteroid Spare, and Drug Retention Rate on Adalimumab Treatment

Based on the use of DMARDs during the first year of follow-up, 29 subjects were classified as belonging to the “ADA+DMARD” group and the other 29 subjects to the “only ADA” group. The remaining 48 patients were cotreated with DMARDs only for a certain period during the first year of ADA follow-up and were therefore excluded from this analysis.

The “only ADA” and “ADA+DMARD” groups were comparable in terms of baseline ocular inflammation: specifically, the proportion of patients with ocular flare in the 12 months before ADA beginning was 79.31% vs. 75.86% (*p* = 1.000). Furthermore, the two groups were comparable in terms of type of ocular involvements (anterior uveitis was present in 55.16 vs. 34.48%; intermediate uveitis associated with either posterior or panuveitis in 34.48% vs. 62.07%; scleritis in 10.34% vs. 3.45%; *p* = 0.118). ME was present in 4 vs. 9 patients (13.79% vs. 31.03%, *p* = 0.231).

As for ocular control, no difference emerged among the two groups (Table 2). In particular, at 6 months of follow-up, percentages of ocular control in the “only ADA” and “ADA+DMARD” groups were 88.00% (22 out of 25) and 72.41% (21 out of 29), respectively (*p* = 0.191). At 12 months, this percentage was 85.00% (17 out of 20) and 76.00% (19 out of 25) in the two groups, respectively (*p* = 0.709). At the last follow-up, ocular control was achieved in 94.44% (17 out of 18) and 100% (13) of subjects.

Considering corticosteroid spare, a significant reduction in median corticosteroid dose was found both in patients with ADA monotherapy and in patients with concomitant DMARD treatment (data not shown). In particular, the median baseline dose of corticosteroids was 12.50 (10-25) mg/day and 6.25 (2.5–15.00) mg/day in the “only ADA” and “ADA+DMARD” groups. At 6 months, the median dose of corticosteroids decreased to 5 (2.5-5) mg/day (*p* < 0.0001) and 5 (0–7.5) mg/day (*p* = 0.004) in the two groups, respectively. At 12 months and at last follow-up (>12 months), the median dose further decreased to 0 (0-5) mg/day (*p* < 0.0001) and 2.5 (0–5) mg/day (*p* = 0.003) in the “only ADA” and “ADA+DMARD” groups.

Focusing on the time of ADA treatment, drug retention rate was more lasting for subjects in the “ADA+DMARD” group (HR of discontinuing ADA treatment of 0.81 (0.30-2.18) for the ADA+DMARDs vs. only ADA group), although the difference was not statistically significant (*p* = 0.682) (Figure 3).

## 4. Discussion

In this retrospective, multicenter real-life study, we confirmed the ocular control of ADA in a large and heterogeneous cohort of patients with noninfectious primary or secondary uveitis. Compared with other retrospective studies, our work included a considerably larger cohort of cases. In particular, Dobner et al. [11] evaluated ADA treatment in 60 patients, Durrani et al. [12] in 32 patients, and Lee et al. [13] in 22 patients. Studies with a larger population were the trials [1, 8], but as compared to the VISUAL-1 and 2 populations, our cohort was mainly composed of subjects with a concomitant systemic inflammatory disorder.

Thus, our results add further evidence on the ocular efficacy of ADA, also in subjects with uveitis secondary to other systemic inflammatory diseases.

Notably, our study showed that ADA is effective also in the long-term follow-up, with more than 90% of the patients with ocular control over a median follow-up of 36 months. Our findings on the long-term ADA control is of importance for clinicians, considering that all other studies had a significantly shorter follow-up [1, 6, 8, 11–13, 16].

Moreover, we observed that patients with low VA at baseline remained stable or improved during ADA treatment, while those with good VA at the start of treatment hardly got worse. A clear description of the final VA based on the different VA at baseline (low, intermediate, and high) was not reported in previous studies. Indeed, at 6 months of follow-up, Durrani et al. [12] showed a VA improvement of at least 20/50 or better in 23% of patients and of 20/200 or better in 60%, while mean VA negligibly decreased. Also in Díaz-Llopis et al.'s study [6], there was an improvement of VA in 21.3% of the patients, stability in 75.4%, and a worsening in 3.3%. These results were partially confirmed by Sheppard et al. [2] in the VISUA1-2 post hoc analysis, suggesting that ADA is associated with a significant improvement of visual functioning in patients with noninfectious uveitis. Our data suggest that the use of ADA should not be limited only to patients with a good VA at the beginning of the treatment but also in those with a more severe VA impairment.

Our study confirmed the corticosteroid-sparing effect of ADA, as reported in registration trials [1, 8] and in retrospective [11–13, 17] and prospective studies [6, 18]. In addition, our data provide additional information on the ability of ADA to reduce prednisone dosage also in the long-term follow-up, thus reducing the exposure to corticosteroids and their side effects in the long term.

Notably, our study showed a good drug retention rate on ADA treatment, as well as a good safety profile. In our study, only 7 patients had side effects requiring treatment interruption. No severe side effects were observed in this large series, in line with both retrospective studies and clinical trials [1, 8].

Of particular relevance, we found that our results on ADA ocular control and on corticosteroid-sparing effect were not influenced by the concomitant use of DMARDs during the first year of follow-up. Similarly, there was no difference in the time of treatment among patients receiving ADA alone or in association with DMARDs. Unlike other inflammatory diseases such as rheumatoid arthritis [18, 19] or juvenile rheumatoid arthritis [20, 21], in uveitis, ADA survival did not appear to be influenced by the concomitant use of DMARDs. These results support and parallel those of Cordero-Coma et al. [22]. Indeed, they did not find any protective effect of concomitant immunosuppression in the formation of anti-ADA antibodies and any difference in the time of treatment in patients with noninfectious primary or secondary uveitis treated with ADA. Our data could be influenced by the quite high number of BS patients among the subjects of our cohort. Indeed, it has already been shown that the time of treatment of ADA is not affected by adding DMARDs in BS patients [23–25]. Notably, these data suggest that adding DMARDs in noninfectious primary or secondary uveitis does not seem useful to reach a longer time of treatment [26–29].

The main limitation of this study is certainly its retrospective nature. However, to date, it is one of the largest studies on the efficacy of ADA for the treatment of noninfectious primary or secondary uveitis in a real-life setting, with a long-term follow-up, and the only one comparing ADA monotherapy vs. ADA associated with DMARDs.

## 5. Conclusions

Our results showed the long-term effective control of ADA also as monotherapy in patients with noninfective uveitis, either primary or secondary to systemic disorders. Based on our results, ADA can be effectively used in subjects with low VA since it proved to effectively preserve or even improve VA. Moreover, our findings suggest that the concomitant use of DMARDs does not seem to provide any additional benefit to ADA alone in terms of ocular control, reduction of steroid dose, and time of treatment.

These data add important suggestions to clinicians for the daily management of patients with noninfectious primary or secondary uveitis in the real-world setting.

## Figures and Tables

**Table 1:** Baseline demographic and clinical characteristics.

	Characteristics *n* (% out of 106)
Sex	
Men	47 (44.34)
Women	59 (55.66)
Median age (IQR) at onset of uveitis, years	29.00 (20.50-40.00)
Associated disease	
No (idiopathic uveitis)	12 (11.32)
Yes	94 (88.68)
Behçet syndrome	66 (62.26)
Other (JIA, PsA, AS, VKH, and IBD)	28 (26.42)
Median follow-up, months	36.02 (16.27-59.17)
Median duration of uveitis at ADA beginning (IQR), years	4.00 (1.00-11.00)
Type of uveitis	
Anterior	45 (42.45)
Posterior and/or panuveitis (and/or intermediate)	51 (48.11)
Unilateral	42 (39.62)
Bilateral	64 (60.38)
Previous treatment	
None	17 (16.04)
Only synthetic DMARDs	59 (55.66)
Only biologics	7 (6.60)
Synthetic DMARDs and biologics	23 (21.70)
Baseline treatment	
None	31 (29.25)
Only synthetic DMARDs	53 (50.00)
Only biologics	7 (6.60)
Synthetic DMARDs and biologics	4 (3.77)
Missing	11 (10.38)
Ocular flare	
In the 12 months before ADA treatment	76 (71.70)
At baseline	70 (66.04)

ADA: adalimumab; AS: ankylosing spondylitis; DMARDs: disease-modifying antirheumatic drugs; IBD: intestinal bowel disease; JIA: juvenile idiopathic arthritis; PsA: psoriatic arthritis; VKH: Vogt-Koyanagi-Harada.

**Table 2:** Ocular control of adalimumab (ADA) therapy, overall and stratified according to the concomitant use of DMARDs.

	Observed	6 months	12 months	>12 months
Overall, *N* (%)				
N. obs.	106	92	78	55
Ocular control	—	77 (83.7)	65 (83.3)	52 (94.6)
No ocular control	—	15 (16.3)	13 (16.7)	3 (5.5)

Stratified according to reason for ADA beginning:				
Ocular (+/- systemic)				
N. obs.	76	65	56	36
Ocular control	—	54 (83.1)	45 (80.4)	33 (91.7)
Systemic				
N. observed	30	27	27	19
Ocular control	—	23 (85.2)	23 (90.9)	19 (100.0)

Stratified according to DMARD cotreatment in the first year:				
Only ADA group, *N* (%)				
N. obs.	29	25	20	18
Ocular control		22 (88.0)	17 (85.0)	17 (94.4)
ADA+DMARD group, *N* (%)				
N. obs.	29	29	25	13
Ocular control		21 (72.4)	19 (76.0)	13 (100.0)
*p* value^∗^		*0.191*	*0.709*	*n.c.*

^∗^ *p* value from the comparison of proportions of ocular control in the “only ADA” vs. “ADA+DMARD” groups; ADA: adalimumab; DMARDs: disease-modifying antirheumatic drugs; n.c.: not calculable.

## Data Availability

The data used to support the findings of this study are available from the corresponding author upon request.
